# Dental Chair

**Published:** 1867-08

**Authors:** 


### DENTAL CHAIR.

We have had in use for some time an operating chair, invented and manufactured by Dr. I. A. Salmon, of Boston. We regard it as one of the best chairs in use.

The body of the chair has a backward, forward and lateral movement. The arrangement for the head-rest gives any desirable position. The chair is especially to be commended for the permanency of its machinery, and accuracy of its movements, and fixedness in any position in which it may be placed.

A change of position is easily effected while the patient is in the chair. The footboard is attached to and moves with the chair. Taken as a whole, we think the chair will answer all the requirements of a Dental chair most fully.

They may be obtained through any of the Depots.

